# Sub-micron level investigation reveals the inaccessibility of stabilized carbon in soil microaggregates

**DOI:** 10.1038/s41598-018-34981-9

**Published:** 2018-11-14

**Authors:** Pavithra S. Pitumpe Arachchige, Ganga M. Hettiarachchi, Charles W. Rice, James J. Dynes, Leila Maurmann, Jian Wang, Chithra Karunakaran, A. L. David Kilcoyne, Chammi P. Attanayake, Telmo J. C. Amado, Jackson E. Fiorin

**Affiliations:** 1 Department of Agronomy, Kansas State University, Manhattan, Kansas 66506 USA; 2 Canadian Light Source, Saskatoon, Saskatchewan S7N2V3, Canada; 3 Department of Chemistry, Kansas State University, Manhattan, Kansas 66506 USA; 4 Present Address: Kansas Department of Health and Environment, 6810 SE Dwight Street, Topeka, KS 66620 USA; 5 Advanced Light Source, Berkeley, California, 94720 USA; 6 Present Address: Department of Soil Science, University of Peradeniya, Peradeniya, 20400 Sri Lanka; 7 Federal University of Santa Maria, Santa Maria, Rio Grande do Sul, 97105-900 Brazil; 8 CCGL Tec and University of Cruz Alta, Rio Grande do Sul, 98005-970 Brazil

## Abstract

Direct evidence-based approaches are vital to evaluating newly proposed theories on the persistence of soil organic carbon and establishing the contributions of abiotic and biotic controls. Our primary goal was to directly identify the mechanisms of organic carbon stabilization in native-state, free soil microaggregates without disrupting the aggregate microstructure using scanning transmission x-ray microscopy coupled with near edge x-ray absorption fine structure spectroscopy (STXM-NEXAFS). The influence of soil management practices on microaggregate associated-carbon was also assessed. Free, stable soil microaggregates were collected from a tropical agro-ecosystem in Cruz Alta, Brazil. The long-term experimental plots (>25 years) comparing two tillage systems: no-till and till with a complex crop rotation. Based on simultaneously collected multi-elemental associations and speciation, STXM-NEXAFS successfully provided submicron level information on organo-mineral associations. Simple organic carbon sources were found preserved within microaggregates; some still possessing original morphology, suggesting that their stabilization was not entirely governed by the substrate chemistry. Bulk analysis showed higher and younger organic carbon in microaggregates from no-till systems than tilled systems. These results provide direct submicron level evidence that the surrounding environment is involved in stabilizing organic carbon, thus favoring newly proposed concepts on the persistence of soil organic carbon.

## Introduction

The increasing concentration of carbon dioxide (CO_2_) in the atmosphere has stimulated a wide array of research exploring mitigation options to counteract climate change. One area with significant potential to reverse this trend, proposed by the Intergovernmental Panel on Climate Change (IPCC), is carbon (C) sequestration in soils/vegetation through agriculture, forestry, and other land uses (AFOLU)^1^. A soil’s capacity as a C sink depends on the initial level of soil organic carbon (SOC), soil characteristics, climate, and management^2^. Mechanisms of SOC sequestration/stabilization and soil aggregation have been debated for decades, however newly proposed concepts have emphasized that SOC persistence is not solely a molecular property but is greatly influenced by the surrounding environment as well^3^. Recent arguments have supported a soil continuum model, highlighting how inaccessibility to microbiota and soil mineralogy affect SOC stabilization^4^. Direct evidence-based approaches are essential to validating these newly proposed concepts^3,4^ with a tremendous need for detailed studies on mechanisms in aggregate dynamics linked with C sequestration^5^ and SOC stabilization in nano- to micrometer-sized microaggregates^6^.

Studies on SOC stabilization have evolved from making observations based on the extraction of organic materials in soil aggregates^7^ to direct observation of soil C stabilization mechanisms by various high-resolution spectroscopic techniques^8^. More recent advancement in this area includes coupling spatially resolved *in situ* imaging with spectroscopic techniques to capture spatial relationships and soil organic matter (SOM) heterogeneity. Studies employing techniques that directly probe the underlying mechanisms driving carbon associations are scarce with investigations attempting to preserve soil aggregate microstructure being even more rare^8,9^ (likely as a result of the tedious sample preparation process required).

Scanning transmission x-ray microscopy coupled with near edge x-ray absorption fine structure spectroscopy (STXM-NEXAFS) is a powerful technique that can image and obtain chemical information in micrometer-sized soil samples at nanometer-scale resolution with minimal disturbance to the specimen^10^. This technique generates element-specific component maps for a broad range of biologically important elements (i.e., C, N, O, P, and S), alkaline metals (i.e., Na, Ca, K, Mg), first row transition metals (i.e., Mn, Fe), Al, and Si^11^ and has been used by many researchers in the recent past to study soil C and mineral associations^8,9,12–14^. Stuckey *et al*. (2017)^10^ has identified this technique as the greatest in elucidating organo-mineral interactions. Studying major elements in soil minerals (Ca, Fe, Al, and Si) is useful along with C as the chemical forms of these minerals can be related to SOC stabilization^8^. Interactions with mineral surfaces and formations of organo-mineral complexes are known to be the primary mechanisms that control the stabilized nature of SOC^3,15^. Clay minerals and iron/aluminum oxy(hydr)oxides are considered as the most important microaggregate forming materials in soils^16^. These minerals are bound together through physicochemical and chemical interactions involving natural gluing and cementing agents^16^, such as oxides, hydroxides, and oxyhydroxides of Fe, Mn, Al, and Si, aluminosilicates, and carbonates. The primary goal of this study was to search for direct evidence of the mechanisms of SOC stabilization using STXM-NEXAFS whilst incurring minimal disturbance to the original aggregate microstructure. These observations were then supported by appropriate bulk chemical analyses (total organic C/TOC, ^13^C nuclear magnetic resonance/NMR, and high-performance liquid chromatography/HPLC) to reveal the effect of long-term management practices on microaggregate-associated carbon.

## Results

### Carbon spectromicroscopy

**Figure 1:**
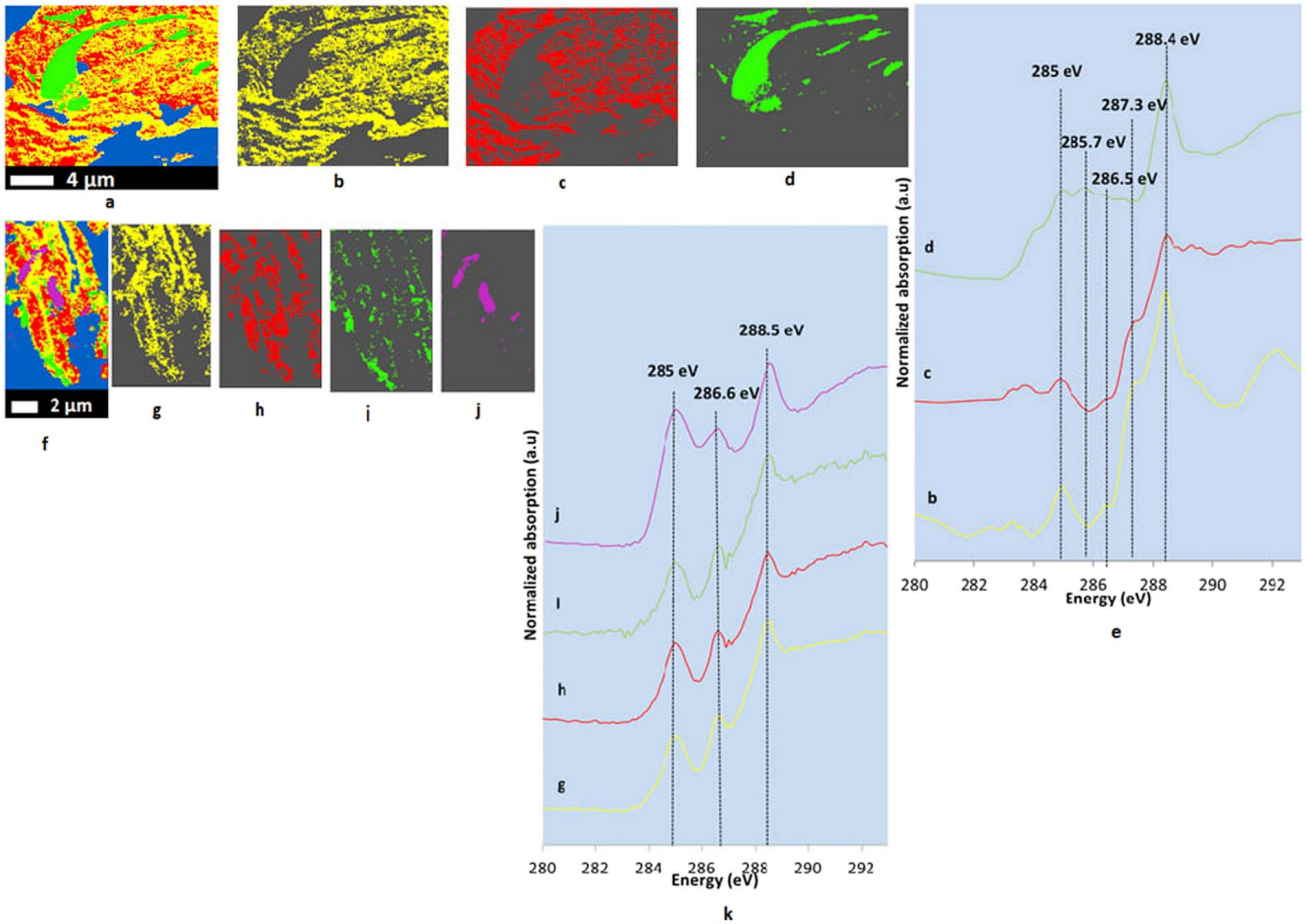
Cluster indices map of C (**a**), individual cluster images (**b–d**), and C K-edge (1 s transitions) NEXAFS spectra (**e**) representing individual cluster images of an NTR 100-nm thin section (20 µm × 15 µm); and C cluster indices map (**f**), individual cluster images (**g–j**), and C K-edge (1 s transitions) NEXAFS spectra (**k**) representing individual cluster images of a CTR 100-nm thin section (8 µm × 20 µm). NTR represents no-till, complex crop rotation. CTR represents conventional till, complex crop rotation. Blue represents empty spaces and areas with high optical density. Individual cluster images with different colors represent the areas with similar spectral properties.

**Figure 2:**
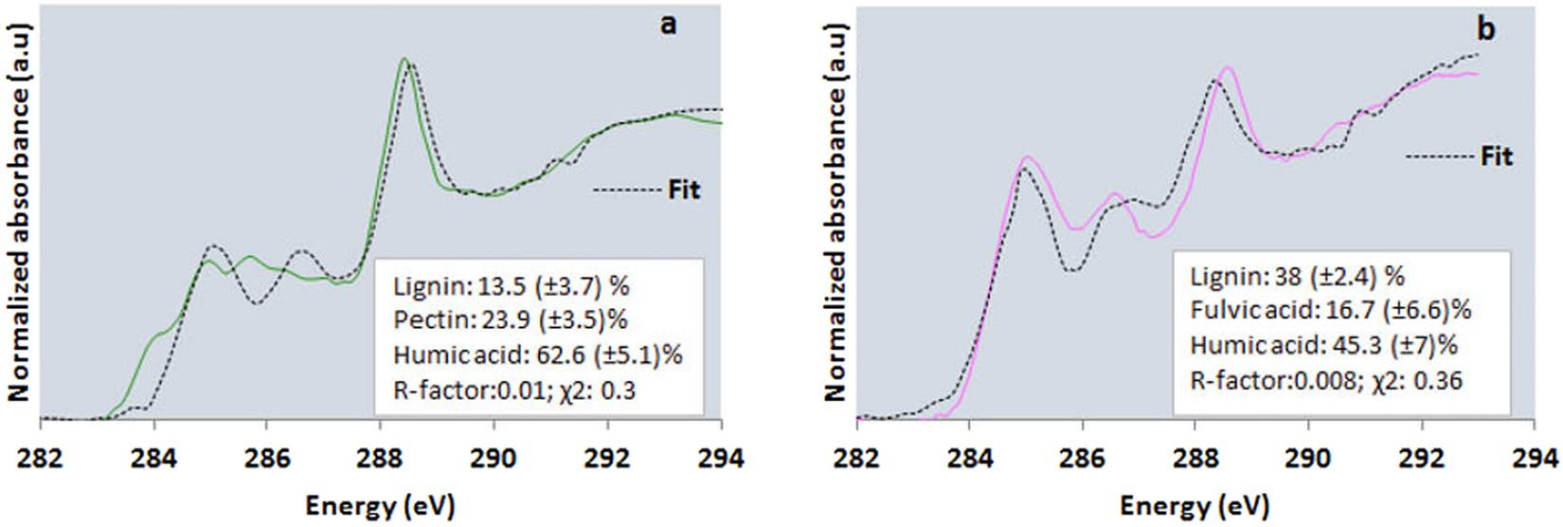
Linear combination fitting (black dotted lines) of C K-edge (1 s transitions) NEXAFS spectra of clusters representing preserved features of NTR (**a**) and CTR (**b**) 100-nm thin sections. NTR represents no-till, complex crop rotation. CTR represents conventional till, complex crop rotation.

Carbon STXM-NEXAFS spectroscopy showed that SOC distributed heterogeneously (Fig. 1a,f, and Supplementary Fig. S1a), inside what appeared to be protective micro- and nano-casings that are made of various minerals such as aluminosilicates, Fe oxyhydroxides, Ca phosphates etc. Carbon K-edge NEXAFS spectra of both NTR and CTR thin sections (Fig. 1e,k) showed resonance peaks (identified from published literature, Supplementary Table S7), representing aromatic ring structures (284.9-285.5 eV)^17,18^, phenolic C/ketonic C (285.3–285.7 eV/286.5–286.7.1 eV)^8^, and carboxylic C (287.8–288.8 eV)^19^. In addition, a shoulder occurred around 287.3 eV in NTR (Fig. 1e; red and yellow spectra), indicating aliphatic C and imidazol ring structures^19^. A less intense peak resembling carbonate C (290.3 eV)^20^ was also present. In NTR, spectrum d (Fig. 1e; green spectrum) is unique compared to b and c spectra (Fig. 1e; red and yellow spectra), suggesting the area represented by cluster d (Fig. 1d) is chemically distinct from other areas. In CTR, spectra j (Fig. 1k; purple spectrum) is different from other spectra (Fig. 1k; yellow, red, and green spectra). Cluster indices maps of both 100-nm thin sections (NTR and CTR) and 800-nm thin section of NTR revealed preserved unique features (spatially distinct hotspots, where OC was appeared in agglomeration) with distinctive C chemistries (Fig. 1d,j, and Supplementary Fig. S1c). In the NTR 100-nm thin section, the preserved feature (Fig. 1d) resembled a portion of a root hair. All spectra (Fig. 1e,k) showed carboxylic C around 288.4 eV and indicated a relative dominance of carboxylic C in both 100-nm thin sections (Supplementary Tables S1 and S2). The root hair-like structure in the NTR thin section (Fig. 1d) showed relatively high phenolic C (≃25%), low carboxylic C (≃55%), and negligible aliphatic C compared to the surrounding area (Supplementary Table S1). Preserved unique features in the CTR thin section (Fig. 1j) showed high aromatic C (≃35%) and low carboxylic C (≃37%) compared to the surrounding area (Supplementary Table S2). To confirm our supposition about the precursors of these preserved features (i.e., root hair-like structure or chemically distinctive from the C in surrounding regions), linear combination fitting of C K-edge NEXAFS spectra was carried out (Fig. 2). The root hair-like structure in the NTR thin section (Fig. 1d) was composed of 13.5% lignin, 23.9% polygalactouronic acid (pectin), and 62.6% humic acid, whereas the two features in the CTR thin section (Fig. 1j) showed 38% lignin, 16.7% fulvic acid, and 45.3% of humic acid (Fig. 2). We used the Suwannee River fulvic acid and humic acid as two additional model compounds to represent the fragments from decomposed plant matter. They are high in aromatic carbon and low in nitrogen, owing to their higher plant precursor materials such as lignin^21,22^. In both these cases, removal of humic acid and fulvic acid standards resulted in significantly poorer fits.

### Calcium, nitrogen, iron, aluminum, and silicon spectromicroscopy

**Figure 3:**
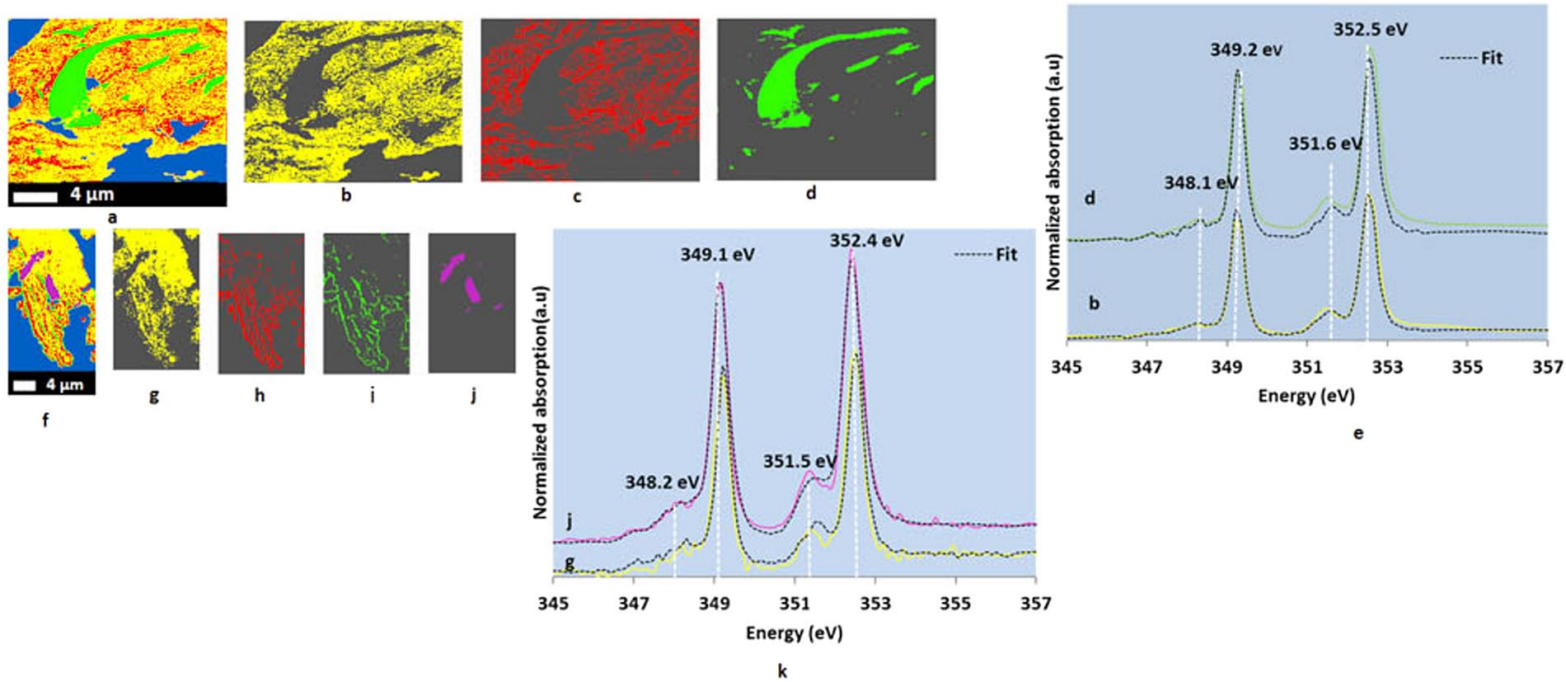
Calcium cluster indices map (**a**), individual cluster images (**b-d**), and Ca Ca L-edge (2p transitions) NEXAFS spectra (**e**) representing individual cluster images of an NTR 100-nm thin section (20 µm × 15 µm); Ca cluster indices map (**f**), individual cluster images (**g**–**j**), and Ca L-edge (2p transitions) NEXAFS spectra (**k**) representing individual cluster images of a CTR 100-nm thin section (8 µm × 20 µm). Only significant fittings (black dotted lines) are displayed (**e** and **k**). NTR represents no-till, complex crop rotation. CTR represents conventional till, complex crop rotation. Blue represents empty spaces and areas with high optical density. Individual cluster images with different colors represent the areas with similar spectral properties.

In the Ca L-edge NEXAFS spectra, two well-resolved peaks (349.2 eV and 352.5 eV) were observed with less intense crystal field peaks near 348.2 eV and 351.5 eV (Fig. 3e,k). Less intense crystal field peaks indicated the amorphous nature of Ca minerals^23^. Preserved features in all thin sections (100 nm and 800 nm) exhibited a distinctive Ca composition (Fig. 3d,j, and Supplementary Fig. S2c). Linear combination fitting of Ca L-edge NEXAFS spectra of NTR 100-nm thin section (Supplementary Table S3) indicated that Ca speciation was mainly dominated by the hydrous calcium dihydrogen phosphate and Ca adsorbed to extracellular polymeric substances (adsorbed Ca_eps), representing Ca associated with microbial products whereas hydrous calcium dihydrogen phosphate, calcium sulphate, and calcite-like minerals were dominated in the CTR thin section (Supplementary Table S4), indicating more biological activities in NTR.

The chemistry of organic N is poorly understood, and one-third to one-half of N is usually categorized as “unknown”^24^. Nitrogen K-edge NEXAFS spectromicroscopy of the CTR thin section exhibited weak signals, attributable to the low concentration of N (data not shown). Nitrogen showed heterogeneous distribution and complex chemistry in the NTR (100 nm) sample (Supplementary Fig. S3). The spectral features indicated the presence of various N containing compounds (Supplementary Fig. S3d) such as aspartic acid, alanine, sulfanilamide, glutamic acid, serine, valine, allantoin etc.^25^. Interestingly, the unique feature identified in the C (Fig. 1a) and Ca (Fig. 3a) cluster indices maps of NTR 100-nm thin section did not appear to possess distinctive N chemistry and was thus not observed (Supplementary Fig. S3a).

**Figure 4:**
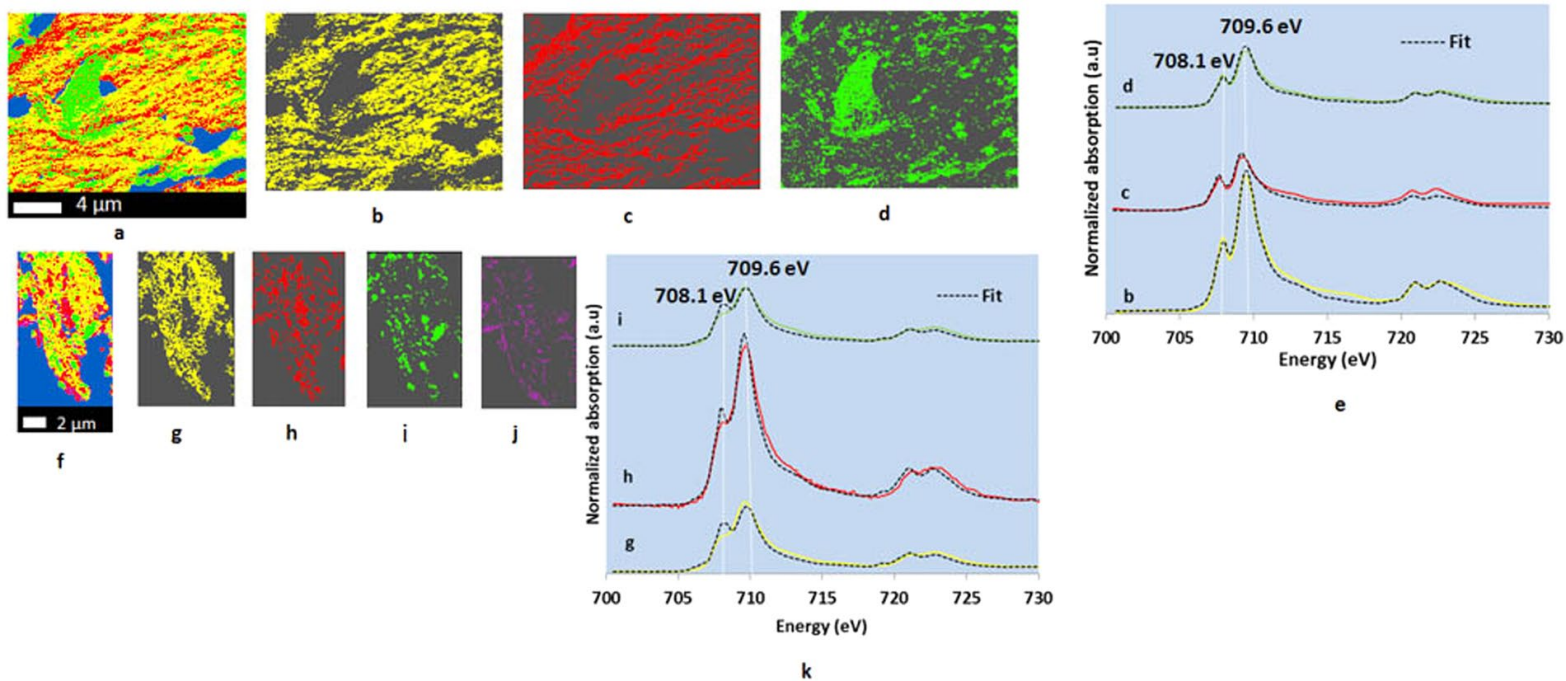
Iron cluster indices map (**a**), individual cluster images (**b**–**d**), and Fe L-edge (2p transitions) NEXAFS spectra (**e**) representing individual cluster images of an NTR 100-nm thin section (20 µm × 15 µm); Fe cluster indices map (**f**), individual cluster images (**g**–**j**), and Fe L-edge (2p transitions) NEXAFS spectra (**k**) representing individual cluster images of a CTR 100-nm thin section (8 µm × 20 µm). Only significant fittings (black dotted lines) are displayed (**e** and **k**). NTR represents no-till, complex crop rotation. CTR represents conventional till, complex crop rotation. Blue represents empty spaces and areas with high optical density. Individual cluster images with different colors represent the areas with similar spectral properties.

Iron L-edge NEXAFS spectra showed multiple peaks at Fe L_3_- (around 708.1 and 709.6 eV) and L_2_- (around 721.1 and 722.5 eV) edges (Fig. 4e,k). Spectral shape at L_3_ 2p_3/2_ reveals Fe oxidation status; the peak at 708.1 eV dominates for Fe^2+^ species, while 709.6 eV is stronger when more Fe^3+^ species are present^26,27^. Linear combination fittings of Fe L-edge NEXAFS spectra for both 100-nm thin sections (NTR and CTR) indicated the presence of Fe^2+^/Fe^3+^ mixed minerals (maghemite, magnetite, and ferric phosphate), with Fe^3+^ being the dominant form (Supplementary Tables S5 and S6). Additionally, NTR (Supplementary Table S5) had goethite and Fe(II) hydroxycarbonate whereas NTR (Supplementary Table S6) had ferrihydrite-like minerals. Cluster indices maps identified, to an extent, a distinctive Fe chemistry in some preserved structures suggesting possible chemical stabilization of OC (Fig. 4d and Supplementary Fig. S4c).

Aluminum K-edge NEXAFS spectra of NTR (800 nm) indicated a dominance of kaolinite and gibbsite-like minerals (Supplementary Fig. S5e). Additionally, the preserved features of 800-nm thin section of NTR (Supplementary Fig. S5c; red spectrum of Supplementary Fig. S5e) indicated an association with pyrophyllite-like minerals^28^. This thin section also exhibited an envelope (lining) with distinctive Si chemistry around the preserved features (Supplementary Fig. S6c) indicating physical protection. Some forms of Si minerals (such as quartz) were found in several places (Supplementary Fig. S6d). To some extent, Al and Si individual cluster images (Supplementary Figs S5c and S6c) were spatially correlated to preserved features (Supplementary Fig. S1c), signifying organo-mineral interactions. Due to limited beamtime, CTR 800-nm thin section was not studied in detail.

### Contrast maps and elemental correlations

**Figure 5:**
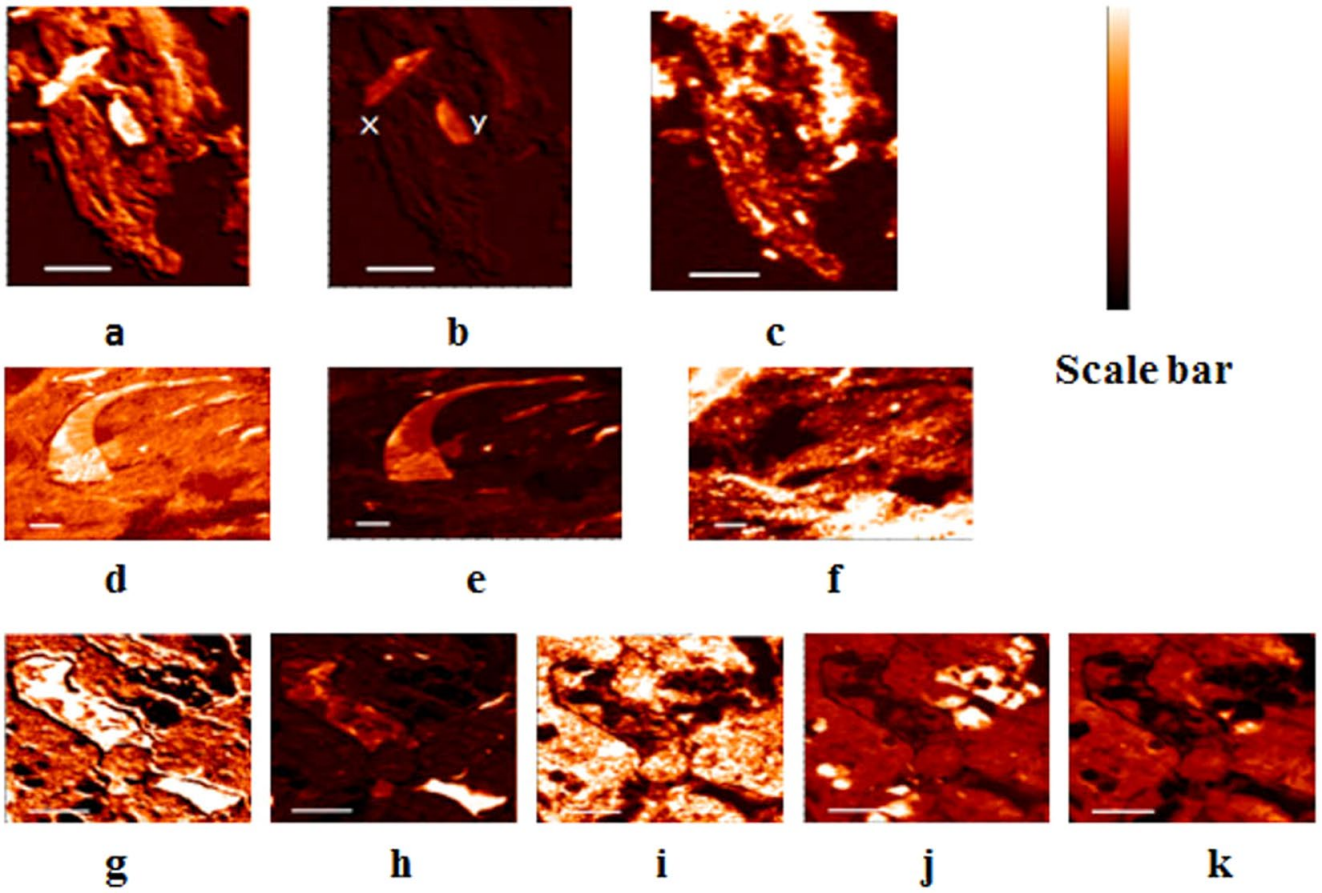
Chemical contrast maps of carboxylic C (**a**), Ca (**b**), and Fe (**c**) of a CTR 100-nm thin section (preserved features are denoted with x and y). Carboxylic-C (**d**), Ca (**e**), and Fe (**f**) contrast maps of an NTR 100-nm thin section. Carboxylic C (**g**), Ca (**h**), Fe (**i**), Si (**j**), and Al (**k**) contrast maps of an NTR 800-nm thin section. High concentration of each element is denoted by the lighter colors of the scale bar.

Contrast maps provided information on spatial distribution and co-existence of the studied elements. Carbon (Fig. 5a,d and g) and Ca (Fig. 5b,e and h) contrast maps clearly indicated a higher concentration of C/Ca in the preserved features than the surrounding material. The co-existence of C and Ca may not be solely due to CaCO_3_, but also due to organic C-Ca bridging and this was confirmed by the weak carbonate peak in C K-edge NEXAFS spectra (Fig. 1e,k). Co-existence of C and Ca (Fig. 5d,e) was evident in the NTR (100 nm) thin section (Pearson’s correlation coefficient = 0.95). Moreover, Fe contrast maps (Fig. 4c,f and i) indicated heterogeneous distribution with some concentrated areas, and distinct C/Fe co-existence in certain areas of the CTR 100-nm thin section (Fig. 4a,c). All preserved features showed relatively low concentrations of Fe, possibly due to their more plant-derived and less weathered nature. Silicon and Al co-existed (Pearson’s correlation coefficient = 0.83) in certain areas of the NTR (800 nm) thin section, indicating the presence of aluminosilicates (Fig. 4j,k) whereas Fe/Al co-existed showing the presence of mixed oxides (Fig. 4I,k). Minerals containing Si (Fig. 5j), with no intimate association with Fe (Fig. 5i) and Al (Fig. 5k), were found in certain areas of the NTR (800 nm) thin section and this can be an indicative of quartz (SiO_2_) particles (Fig. 5j).

### Bulk chemical analysis

The microaggregate-associated OC content in NTR was 3.2% and 1.8% in CTR. The ^13^C nuclear magnetic resonance spectra of humic acid extracted from the microaggregates (NTR and CTR) indicated intense peaks of carboxylic, aromatic, O-alkyl, and alkyl C (Supplementary Fig. S7). Carbohydrate (61–83 ppm) and amino acid (47–60 ppm) peaks were identified in both NTR and CTR^29^. Detailed information on calculation of the ratio of hydrophilic C to hydrophobic C (HIL/HOB) is stated in the method section. An increase in the HIL/HOB ratio coincides with an increase of the degree of maturity in humic acids^30^. Also, the HIL/HOB ratio reflects the degree of transformation of SOM^30^. Comparing the HPLC and NMR analyses of extracted humic acid indicated a low hydrophilic/hydrophobic (HIL/HOB) ratio in NTR (Tables 1 and 2), suggesting lesser maturity of organic C than in CTR.

**Table 1:** Percentages of different C functional groups in humic acid extracted from microaggregates (150–250 µm), determined by ^13^C nuclear magnetic resonance spectroscopy (Supplementary Fig. S7).

	Carboxyl	Aromatic	O-alkyl	Alkyl	HIL	HOB	HIL/HOB ratio
	%						
NTR	7.6	50.6	21.3	20.5	28.9	71.1	0.41
CTR	10.4	35.2	31.2	23.2	41.6	58.4	0.71

Four regions: carboxyl C (220–160 ppm), aromatic C (160–100 ppm), O-alkyl C (110–45 ppm), and alkyl C (45–10 ppm). Aromatic and alkyl C represent hydrophobic (HOB) fraction. Carboxylic and O-alkyl represent hydrophilic (HIL) fraction. NTR represents no-till, complex crop rotation. CTR represents conventional till, complex crop rotation.

**Table 2:** Percentages of hydrophilic (HIL) and hydrophobic (HOB) C in humic acid extracted from microaggregates (150–250 µm), determined by high performance liquid chromatography.

	Hydrophilic[HIL]	Hydrophobic[HOB]	HIL/HOB ratio
	%		
NTR	59.8	40.2	1.5
CTR	71.3	28.7	2.5

NTR represents no-till, complex crop rotation. CTR represents conventional till, complex crop rotation.

## Discussion

Our study verified the ability of non-invasive STXM-NEXAFS spectroscopy to obtain submicron level multi-elemental information to understand organo-mineral associations. Inability to analyze soil samples in replicates and use of small quantities of samples in STXM-NEXAFS spectroscopy are often questioned for its reproducibility and scalability^11^. To overcome those challenges, soil scientists usually prepare composite samples by mixing randomly taken multiple soil samples from the area of interest; and grinding and sieving soils to make representative homogeneous samples^11^. In our experiment, we obtained a representative soil sample by mixing randomly taken 15–20 small soil samples from the field plot. Our goal was to analyze undisturbed microaggregate thin sections with preserved aggregate architecture, so grinding of soils was not possible. For our study, free microaggregates (i.e. not occulded in macroaggregates; 150–250 µm in size) were selected based on their potential in bearing stabilized C. The largest pool of stable organic C is believed to be in soil microaggregates^31,32^ and free microaggregate-associated C possesses a slower turnover rate compared to macroaggregate-associated C^33^. Part of this may be due to the fact that microaggregates can survive tilling operations more than macroaggregates^31,34^. The strength of microaggregates is gained through associations with resistant binding agents^16,31,35^. Associations of bacterial residues/hyphal debris and clay microstructures form silt-sized microaggregates (20–53 µm) while fungal and plant debris form large microaggregates (53–250 µm)^36^.

Carbon spectromicroscopy indicated that C located inside nano- and micro-casings (Fig. 1a,f, and Supplementary Fig. S1a) in soil microaggregates, exerting strong physicochemical protection. A recent study identified distinct microstructural domains that regulate soil C storage and highlighted that those domains form by self-organizing the mineral and organic components, forming mineral assemblages^37^. Also, the presence of an organic core (preserved unique features) in these thin sections supports the theory that microaggregates may form around OM particles^33,38,39^. The heterogeneity of SOC in this system was due to continuous and diverse C inputs and the climate that led to different degrees of decomposition^40^. Combinations of continuous C input and relatively low physical disturbances resulted relatively slower C decomposition rates, in turn giving high C storage and younger C in NTR compared to CTR^40^. Long-term, diversified crop rotations in this system could have enhanced the quality, quantity, and chemical diversity of residues and impacted soil microbiota, creating beneficial effects on SOM and soil fertility^41^. The wet subtropical agroecosystem in this study had climatic conditions (high temperature and precipitation) favorable to high microbial activity throughout the year^42^, leading to reduced stable SOC^43^. The complexity in aggregation and organo-mineral assemblages also led to variabilities in levels of decomposition. High C input in NTR system is concentrated in a smaller soil volume (top soil) compared to CTR where the C input gets continuously mixed and diluted into a larger volume of soil due to tilling. Further, in NTR, the possibility of incorporating roots into aggregates can be high due to the abundance of root growth in the undisturbed top soil, giving more opportunity for continuous and steady flow of useable (labile) C input.

This study, using spectromicroscopy, provided evidence on carbon storage in this long-term field experiment with a variety of C inputs and contrasting soil disturbances. It has been well established that soil mineralogy dominates aggregate stabilization in Oxisols^32,44^ and this study provided evidence how young C associates with soil minerals though various organo-mineral interactions forming microaggregates. Organic matter associates with soil minerals via various mechanisms including ligand exchange, polyvalent cation bridges, and weak interactions like van der Waal’s forces and H-bonding^45^. The presence of preserved, micron-sized, partly weathered OC particles (Fig. 1d,j), with original morphology and distinctive C chemistry, exemplified the strength of C stabilization mechanisms. Further, those preserved unique features may not be necessarily chemically recalcitrant^46^. Spectra of the preserved structures (NTR and CTR 100-nm thin sections) had a peak at 288.4/288.5 eV (Fig. 1e/green spectra and 1k/purple spectra) indicating either the presence of carboxyl C^8^, amide functional groups^8^, and/or amino acids with side chains containing carboxylic and amine groups^47^. Microbial-derived C always has a characteristic absorption feature at 288.2 eV, indicating the presence of peptide bonds^48,49^. Absence of a peak at 288.2 eV and the components (lignin and pectin) observed from linear combination fitting (Fig. 2) confirmed the plant-derived nature of those preserved structures. Further, phenolic C in preserved root-hair like structure (Supplementary Table S1) indicated plant-derived C from lignin degradation^50^. The presence of humic and fulvic acid like components (via linear combination fitting) in the preserved structures (Fig. 2) could be indicating a partial decomposition (i.e. different from known biomolecules).

The high clay content (52% clay) in this soil suggests most pores are inaccessible to microbes because diameters of pore necks are usually smaller than 0.2 µm in clayey soils^51^. Therefore, protection was most likely achieved through combinations of spatial and kinetic constraints on microbial accessibility and OC degradability^52^. The presence of easily decomposable^53^ aliphatic C in the NTR thin section (Supplementary Table S1), as observed in the spectromicroscopy study, could be linked with strong stabilization mechanisms (slow OC decomposition rates and strong protection mechanism). Aliphatic C indicates the presence of cell wall lipids^54^ which can be preserved through hydrophobic bonding of OC and minerals^54^, and also favorably adsorbed by clay minerals such as kaolinite and montmorillonite^55^. Negligible aliphatic C in the CTR thin section could be an indication of relatively high decomposition rates, where the SOC become highly aromatic, forming polycondensed rings^56^.

The soil microbial population may also be participating heavily on soil C stabilization in these agroecosystems. The contribution of soil microbes in producing chemically diverse, stable SOC has been brought forward by researchers with direct evidence^57^. Their study showed that microbial communities impact on SOM accumulation more than the clay mineralogy. In top soil, decomposed plant materials interact with metal oxides whereas in deeper layers, associations of metal oxides and OM exposed to microbial turnover are dominant^58^. Microbial efficiency-matrix stabilization hypothesis suggests that the microbial use effectiveness is governed by the stabilization through organo-mineral associations^59^. Nitrogen rich microbial products, due to their positively charged functional groups, favorably associate with mineral surfaces compared to C-rich moieties enhancing the storage of SOM^60^. The heterogeneous and complex distribution of N-based compounds observed in the NTR thin section (100-nm) could be partly indicating microbial contribution in associating SOM with soil mineral surfaces.

The presence of amorphous Ca minerals (Fig. 3e,k; indicated by less intense crystal field peaks near 348.2 eV and 351.5 eV) enhances building organo-mineral complexes because of high surface area and small size^61^. Our findings provided evidence of the contribution of Ca minerals in preserving OC (Fig. 5a,b,d,e,g and h). Co-existence of C and Ca suggested chemical stabilization of OC by way of electrostatic cation bridges (involving negatively charged siloxane surfaces and/or hydroxyls of aluminosilcates and oxides)^27^ and complexes with metallic/hydroxyl metallic compounds. Moreover, co-existence of C and Fe (Fig. 5a,c) suggested strong polyvalent cation bridges between C and Fe minerals^45^ and/or co-precipitations of OC and Fe^62^, thereby helping stabilize OC. Further, if the decomposition of the root hair was just beginning, the co-existence of C and other elements could be an indicative of the nutrient uptake by the root hair (NTR 100-nm thin section). This remains questionable as the linear combination fitting of the preserved features identified humic and fulvic acid like components, indicating a partial decomposition. Every presented preserved feature showed C/Ca co-existence although all of them may not be root hairs.

The presence of easily degradable amino acid-based compounds in the extracted humic acid from both NTR and CTR free microaggregates (observed via ^13^C NMR) suggested the release of young C likely from physical occlusion during the wet chemical extraction and this observation indirectly supported the STXM-NEXAFS observations. Kaolinite, goethite, and poorly crystalline metal oxyhydroxides (i.e., ferrihydrites), which are high in the studied soil type^63^ (Supplementary Tables S5, S6, and Fig. S5), favor sorbing amino acids^64,65^, and this suggests another possibility for the presence of amino acid-based compounds. The presence of some reduced forms of Fe in both NTR and CTR (Supplementary Tables S5 and S6) could be linked with the Fe^3+^/Fe^2+^ cycling due to continuous availability of root exudates^66^ associated with the crop rotation in this agroecosystem. Metastable Fe (II) hydroxycarbonate (chukanovite), observed in the NTR thin section (Supplementary Table S5), which over time usually transforms to common, thermodynamically more stable Fe oxides in aerated environments^67^, indicated O_2_-depleted microsites due to microbial activity^68^. The high concentration of organic substances^69–71^ and low pH^72^ in this soil (pH 5.1; Table 3) may have favored the formation and stabilization of poorly crystalline Fe precipitates like ferrihydrites through ligand-promoted dissolution, and the OC content subsequently becomes less prone to biodegradation by adsorption and co-precipitation^73^. Soil minerals like goethite (Supplementary Table S5) can bind C compounds via multiple complexations into their mouths of nano-pores, resisting enzymatic attacks^74^. Moreover, the co-existence of Al/Si and C could be due to interactions (such as van der Waal’s forces and hydrophobic bonding) between nonpolar organic molecules/alkyl C/aromatic C and siloxane surfaces of kaolin-group minerals^75^. Iron and Al^3+^ form strong coordination complexes with organic compounds more efficiently compared to Ca^2+^ ^45^. The presence of pyrophyllite-like minerals in the preserved structure (800-nm thin section; Supplementary Fig. S5c) could be an indication of organo-mineral associations as pyrophyllite can sorb organic carbon via van der Waal’s forces and Ca^2+^ bridging^15^. An envelope (lining) with a distinctive Si chemistry around the preserved features observed in NTR 800-nm thin section (Supplementary Fig. S6c) could also take as an evidence of physical protection. All these organic matter-mineral interactions are influenced by the chemistry of both ligand and soil minerals, and the associations are affected by soil pH and ionic strength, creating differences in binding energies^76^.

Bulk analyses showed low OC in tilled free soil microaggregates, highlighting the benefits of no-till, which favor the formation of stable soil microaggregates where C is stabilized via physical protection^32,77,78^. Continuous high C input and less disturbance enhance soil C sequestration/stabilization^79,80^. Supporting this, ^13^C NMR and HPLC showed mature C groups (Tables 1 and 2) in extracted humic acid from tilled microaggregates (i.e., CTR showed higher ratios of hydrophilic C to hydrophobic C than NTR). It should be noted here that the process of “humification” is widely questioned due to lack of proof^4^. Lehmann and Kleber (2015)^4^ and citations there in, proposed SOM is a continuum of progressively decomposing organic compounds. Novel insights on SOC persistence contradict previous beliefs on recalcitrance of input biomass and humic substances^3^. A quick turn over of molecules such as lignin and plant lipids (believed to be persistent in soil) than bulk OM has been reported^81–83^. Further, direct *in-situ* studies have recognized humic substances as molecules with small, simple structures even though they were previously known as large, complex macromolecules^9,84,85^. Humic and fulvic acids are operationally-defined fractions linked with disadvantages such as its inability to truly represent SOM. Further ionization of compounds due to harsh alkaline treatment overestimates their reactivity^4^. Lehmann *et al*. (2008)^9^ reported that spectral signature of native OM was much different from that of the alkaline extracted humic substances. We noticed the presence (via linear combination fitting) of components similar to humic and fulvic acid standards in preserved structures of both NTR and CTR thin sections. In this study we used the characterization of humic acid as a qualitative secondary approach (via ^13^C-NMR) to complement our STXM-NEXAFS work. Although we identified humic acid and fulvic acid like components in preserved structures while fitting our C K-edge NEXAFS spectra with known biomolecules and those two unknowns, our findings do not provide any insights as to their structures or stability. It only suggests some C in preserved structures is different from their precursor plant materials.

**Table 3:** Soil chemical characteristics of 0–5 cm soil layer of NTR and CTR. Adapted from Fabrizzi *et al*.^42^.

	pH	Ca^2+^	K^+^	Bray-P	CEC	Sand	Silt	Clay
		mg kg^−1^			cmol_+_ kg^−1^	%		
NTR	5.1	1004	261	26.8	17.1	25	23	52
CTR	5.1	681	146	18.4	16.2	25	24	51

Overall, stabilization of OC in microaggregates is due to a large array of physicochemical (i.e., chemical and mineralogical) and biological mechanisms as well as management strategies. Microscale findings and direct evidence are useful in building better mechanistic models of soil C stabilization. This study provided direct submicron level evidence of the involvement of ecosystem properties on SOC stabilization and supported the concepts brought forward by Schimidt *et al*.^3^, and Lehmann and Kleber^4^. Further, it shows that a no-till system with complex crop rotation encouraged stabilization of easily degradable organic carbon via either physcial occlusion or intimate association with soil minerals. This study also demonstrated the potential of using a non-invasive spectromicroscopic approach in unraveling submicron level information on soil C stabilization. The cycling of organic matter attracts interest of the researchers from multiple disciplines. Although accessibility is a major constraint, the use of STXM-NEXAFS to study cycling of soil organic matter has the potential to settle the contentious nature of organic matter. Further in our study, ^13^C-NMR and HPLC analyses on extracted humic acids indicated that persistence of soil organic matter does not correlate with its “recalcitrance” (i.e., provided evidence of less “recalcitrant” organic C in no-till system soils). Analysis of extracted humic acids in this study also provided evidence in favor of newly proposed theories on soil carbon stabilization.

## Methods

### Soil characterization

Soil samples were collected from a long-term agricultural experiment (established in 1985) located in Cruz Alta, Rio Grande do Sul, Brazil (28° 33′ S 53° 40′W, 409 m of altitude). Mean annual precipitation was 1,774 mm, and mean annual temperature was 19.2 °C. Soil type was a clayey, kaolinitic, thermic Rhodic Hapludox, enriched with kaolinites and Fe oxides^63^. This field experiment was established as a split-plot randomized block design without replications (main plots: tillage; split plots: crop rotation). There were three levels of crop rotations (based on the level of complexity of crop rotation) and two levels of tillage (no-till and conventional till). We selected the most complex crop rotation (summer and winter crop rotation: wheat/soybean/black oat/soybean/black oat + common vetch/maize/forage radish) with no-till (NTR) and conventional till (CTR) that have led to different levels of aggregation and soil carbon levels^42,63^. These plots were amended with dolomitic lime in 1985 before the study began; dolomitic lime amendments were repeated in 1995 and 2011 at 5 Mg ha^−1^. Soils were sampled to a depth of 0–5 cm in December 2012 using a soil sampling probe (2-cm in diameter) from 15–20 locations of each plot. Moist soils were packed in polypropylene bags to minimize physical damage to aggregates and shipped to Kansas State University, Manhattan, KS, USA. A subfraction was separated and kept frozen at −4 °C.

### Preparation of 100- and 800-nm thin sections

Thin sections were prepared following Solomon *et al*.^8^. Frozen soil was thawed, passed through a 250-μm sieve, and trapped on a 150-μm sieve. Free (not occluded in macroaggregates; around 20 microaggregates) stable microaggregates (150–250 μm) with minimal damage to outer edges were selected under a light microscope (x40). Using a needle (BD microlance needle, Fisher Scientific, USA), microaggregates were placed on a glass fiber filter (Whatman GF/A, 90-nm-diameter, Sigma-Aldrich, USA) positioned on a 7.5-cm-diameter sieve. Microaggregates were saturated with ultra-pure water for 16–18 hrs using a cold-mist humidifier (Vicks® Ultrasonic humidifier, Kaz USA, Inc., USA) with an attached glass chimney directing cold mist onto the filter paper. Once microaggregates were saturated, excess water was drained. Microaggregates were immobilized on sample carriers and flash-frozen using liquid nitrogen. Thin sections were made using a cryo-ultramicrotome (EM UC7/EM FC7, Leica Microsystems Inc., Bannockburn, Illinois, USA) at −55 °C as described below. Thin sections (100 nm) were used for C, Ca, N, and Fe analysis, and 800-nm thin sections were used for Al and Si analysis. A trimming knife (Cryotrim 20, Diatome Ltd., Biel, Switzerland) was used to create a flat smooth surface on the microaggregate; the final cutting was done with a diamond knife (Cryo 35°, Diatome Ltd., Biel, Switzerland) at a cutting speed of 1.2 mm s^−1^ and a cutting angle of 6°. An eyelash probe was used to transfer thin sections onto C-free, copper (Cu) transmission electron microscopy grids impregnated with silicon monoxide (200 meshes, No. 53002, Ladd Research, Williston, Vermont, USA). Sample specimens were stored in a helium atmosphere to prevent oxidation.

### Spectromicroscopy data acquisition and analysis

Data was collected on beamline 5.3.2.2 (polymer STXM) at the Advanced Light Source (ALS), Berkeley, USA^86^, and on 10ID-1 (SM) beamline at the Canadian Light Source (CLS), Saskatoon, Canada^87^. Thin sections (100-nm) of NTR were examined for C, Ca, N, and Fe at the CLS, while thin sections (100-nm) of CTR were examined for the same elements at the ALS. Additionally, an 800-nm thin section of NTR was analyzed at the CLS to obtain further information on Al and Si. The beamline at ALS (250–780 eV) used a bending magnet with an energy resolution of (E/ΔE) ≤ 3,000, and the CLS beamline had a wide energy range (130–2700 eV), which originated in a 75-mm generalized Apple II elliptically polarizing undulator (EPU) with an E/ΔE of 3000–10,000. Data from the CTR thin sections were collected at the ALS in 2014, while NTR thin section data were acquired at the CLS in 2015. Energy ranges of the two beamlines allowed us to examine the K-edge of C and N, as well as the L-edge of Ca and Fe. In addition, the wide energy range of the SM beamline at the CLS allowed collecting information on Al and Si. The monochromators were calibrated using CO_2_ peaks. Peak shifts for Si were corrected using Si_3_N_4_ absorption as the reference. When the energy absorbed by a core electron is equal or greater than the binding energy, an edge is resulted and edges are labelled based upon the shell where the core electron originates from. Excitation of 1 s electrons occurs at the K-edge whereas a 2 s or 2p electrons are excited at an L-edge.

Small areas of thin sections were selected with the following criteria in mind: thickness, presence of morphologically interesting preserved features (i.e., root hairs, coagulated OC sources, etc.), and amount of disturbance (i.e., fewer empty spaces). High-resolution scans were conducted in the selected area at nanometer-scale resolutions and dwell time was modified at particular energy ranges considering absorption edges of elements of interest. For the CTR 100-nm thin section (at ALS), a stack data set for C and Ca was collected with an energy increment of 0.25 eV for the energy range from 275–282 eV (dwell time, DT, 1.222 ms), 0.1 eV from 282.1–300 eV (DT, 1.222 ms), 0.25 eV from 300.25–320 eV (DT, 1.222 ms), and 0.351 eV from 320.35–340 eV (DT, 1.222 ms). A stack data of Fe was collected with an energy increment of 0.5 eV for the energy range of 700–703 eV (DT, 1 ms), 0.1 eV from 703.25–727 eV (DT, 1 ms), and 0.5 eV from 727.5–735 eV (DT, 1 ms). For the NTR 100-nm thin section (at CLS), a stack data set was collected for C, Ca, N, and Fe together with an energy increment of 0.15 eV for the energy range from 280–735 eV (DT, 1 ms). For the 800-nm thin section, a stack data set of Al and Si was collected for the energy range from 1555–1900 eV (DT, 1 ms).

Individual images collected at all energy levels were built into a stack using Stack Analyze 2.6.1 software^88^. Images with uneven intensities were removed from the stack, and the stack was aligned. Principal component and cluster analysis using the PCA GUI 1.1.1 program^89^ identified areas with similar spectral properties. Based on eigen spectra, eigen values, and eigen images^9^, components and clusters were selected for further analysis. Cluster analysis is proven as a successful method for recognizing chemically distinct regions of complex specimens as it can classify the areas into groups considering similar spectral properties and thickness^89^. Different clusters are denoted with different colors. Spectra were normalized using ATHENA^90^. Peaks of C K-edge NEXAFS spectra were identified from published research (Supplementary Table S7). Linear combination fitting was carried out for some C K-edge NEXAFS spectra (only for spectra representing preserved unique features of 100-nm thin sections) and Ca, and Fe spectra of both 100-nm thin sections (NTR and CTR) using ATHENA^90^. For linear combination fitting of C K-edge NEXAFS spectra, aragonite^27^, arbinoxylan^91^, cellulose^91^, DNA^49^, glucan^91^, xylan^91^, lignin^91^, lipid^92^, polygalacturonic acid^91^, polysachcharide/xanthum gum^92^, albumin^49^, Suwannee river fulvic acid standard (IHSS standard; not published), and Suwannee river humic acid (IHSS standard; not published) were used.

For linear combination fitting of Ca L-edge NEXAFS spectra, aragonite^93^, adsorbed Ca_eps (extra polymeric substances)^93^, hydrous calcium dihydrogen phosphate (Sigma-Aldrich,USA; not published), calcite^93^, and calcium sulphate (Sigma-Aldrich, USA; not published) were used. For linear combination fitting of Fe L-edge NEXAFS sepctra, ferrihydrite^94^, goethite^94^, ferric phosphate_dihydrate (Sigma-Aldrich, USA; not published), maghemite^95^, magnetite^95^, and siderite^95^ were used. Nitrogen K-edge spectra of standards^25^ were re-created using Techdig 2.0 software (Supplementary Fig. S3). In addition, Gaussian peak fitting (Supplementary Fig. S8) was used to determine relative proportions of C functional groups of each C spectrum, as they corresponded to individual cluster images from the analysis using ATHENA^90^. An arctangent function for the ionization step at 290 eV and Gaussian peaks representing aromatic, aliphatic, ketonic C/phenolic C, and carboxylic C were used for deconvolution. An arctangent function was fixed at 1.5 eV, and Gaussian peaks were fixed at 0.4 eV of full width at half maximum (FWHM)^96^. Relative percentages of C functional groups representing each transition were determined by setting the area under the Gaussian curve to 100%. The R-factor was optimized to obtain the best fit. The amplitude represented the area under the curve because peak shapes were being unit-normalized. Contrast maps were generated by the difference between the energy of a strong characteristic absorption feature and the energy below the onset of the absorption feature (OD_peak_ − OD_background_). The correlations of Ca/C and Al/Si were determined using ImageJ (http://wwwfacilities.uhnresearch.ca/wcif/imagej/) via the JACoP plugin (https://imagej.nih.gov/ij/plugins/track/jacop.html).

### Bulk soil analysis

Bulk chemical analysis was done as a secondary study to determine qualitative differences in microaggregate-associated C fraction. Bulk chemical analyses were carried out using a free soil microaggregate fraction (150–250 µm). Organic C was determined by dry combustion using a Carlo Erba C/N analyzer (Carlo Erba Instruments, Milan, Italy). Humic acid (HA) was extracted following the International Humic Substances Society method^97^ with some modifications (no hydrochloric acid/hydrofluoric acid treatment) and analyzed using ^13^C NMR (Varian Mercury Spectrometer (400 MHz). Additionally, hydrophilic and hydrophobic properties of humic acid were determined with a liquid chromatograph Gilson system with a DAD detector.

To prepare samples for NMR analysis, humic acid dissolved in NaOH extractant was twice passed through 0.2-µm nylon filters and once through an amberlite (Amberlite IR120 hydrogen form, Sigma-Aldrich, USA) column (2.5 g amberlite per 50 mL of NaOH extractant) with gaseous N_2_ pressure to remove paramagnetic ions^98^. Approximately 20 mg of HA were dissolved in 0.4 mL of 0.3 *M* NaOD/D_2_O solution, mixed well using a vortex, and centrifuged at 3300 rpm^99^. Dialysis was performed to remove salts and chloride ions in the HA extractant before freeze drying. Solution state ^13^C NMR of the freeze-dried HA was conducted on a Varian Mercury spectrometer (400 MHz) working at 100.58 MHz on ^13^C using a 5 mm SW probe. Spectra were obtained by proton broad band decoupling, and samples were run with a 45° pulse and an interpulse delay of 0.5 s. Spectral width was set to 30,000 Hz and 200,000 transients were recorded. Sample temperature was kept at 25 °C. Fourier transform of the resulting data was zero filled to 8,192 data points, and a line broadening of 200 Hz was applied to all spectra. Tetramethylsilane was used as an external chemical shift reference. Areas of aromatic (160–100 ppm) and alkyl C (45–10 ppm) were used to calculate hydrophobicity; O-alkyl (110-45 ppm) and carboxylic (220-160 ppm) areas were used to calculate hydrophilicity of the HAs^100^.

High performance liquid chromatography characterization is based on the amphiphilic properties of HA, where the hydrophilic components are eluted first and then the hydrophobic constituents are fractioned, depending on the strength of their hydrophobic interactions with the hydrophobic matrix. For separation, an Atlantis T3 column (5 µm, 250 × 4.6 mm, 100 A) was used. The mobile phase of deionized water and acetonitrile (flow rate 1 mL min^−1^) used a gradient elution program. HA samples were digested in 0.01 *M* NaOH, at a concentration of 2g L^−1^, for 24 hrs. A 5 µL sample was injected into the column. Chromatograms were analyzed at 254 nm^30^.

## Electronic supplementary material

Supplementary Information

## Data Availability

The data (spectromicroscopy data obtained at the Canadian Light Source, Saskatoon, Canada and Advanced Light Source, Berkeley, CA, USA) that support the findings of the study are available at, https://figshare.com/account/articles/5764650 (ref.^68^).
