# Supplementary material for: Sub-micron level investigation reveals the inaccessibility of stabilized carbon in soil microaggregates
Source: Sci Rep. 2018 Nov 14;8:16810. doi: 10.1038/s41598-018-34981-9 (PMC6235917; doi:10.1038/s41598-018-34981-9)
Supplement: Supplementary file 1 — Supplementary Information [file 41598_2018_34981_MOESM1_ESM.docx]

**Sub-micron level investigation reveals the inaccessibility of stabilized carbon in soil microaggregates**

Pavithra S. Pitumpe Arachchige^1^, Ganga M. Hettiarachchi^1*^, Charles W. Rice^1^, James J. Dynes^2^, Leila Maurmann^3,4^, Jian Wang^2^, Chithra Karunakaran^2^, A. L. David Kilcoyne^5^, Chammi P. Attanayake^1,6^, and Telmo J.C. Amado^7^ and Jackson E. Fiorin^8^

^1^Department of Agronomy, Kansas State University, Manhattan, Kansas 66506, USA. ^2^Canadian Light Source, Saskatoon, Saskatchewan S7N2V3, Canada. ^3^Department of Chemistry, Kansas State University, Manhattan, Kansas 66506, USA. ^4^Kansas Department of Health and Environment, 6810 SE Dwight Street, Topeka, KS 66620, USA. ^5^Advanced Light Source, Berkeley, California 94720, USA. ^6^Department of Soil Science, University of Peradeniya, Peradeniya 20400, Sri Lanka. ^7^Federal University of Santa Maria, Santa Maria, Rio Grande do Sul, Brazil 97105-900. ^8^CCGL Tec and University of Cruz Alta, Rio Grande do Sul, Brazil 98005-970.

Corresponding author: ganga@ksu.edu

**Supplementary Figures**


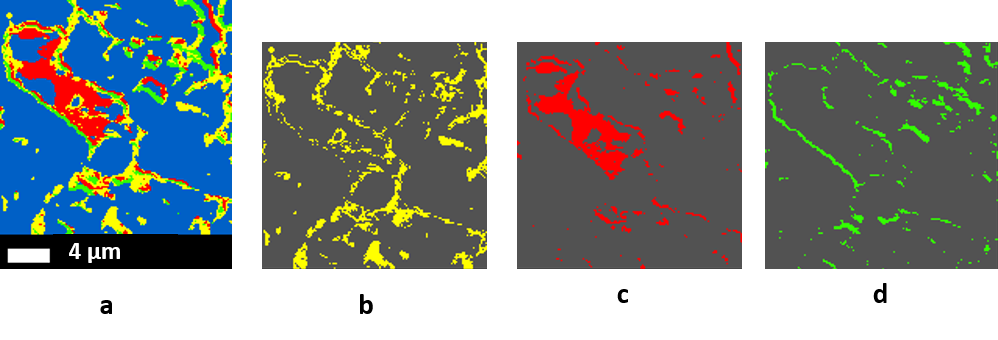


Figure S1. Cluster indices map of C (a) and individual cluster images (b-d) of an NTR 800-nm thin section (22 µmx22 µm). Blue represents empty spaces and areas with high optical density. NTR represents no-till, complex crop rotation. Individual cluster images with different colors represent the regions with similar spectral properties.


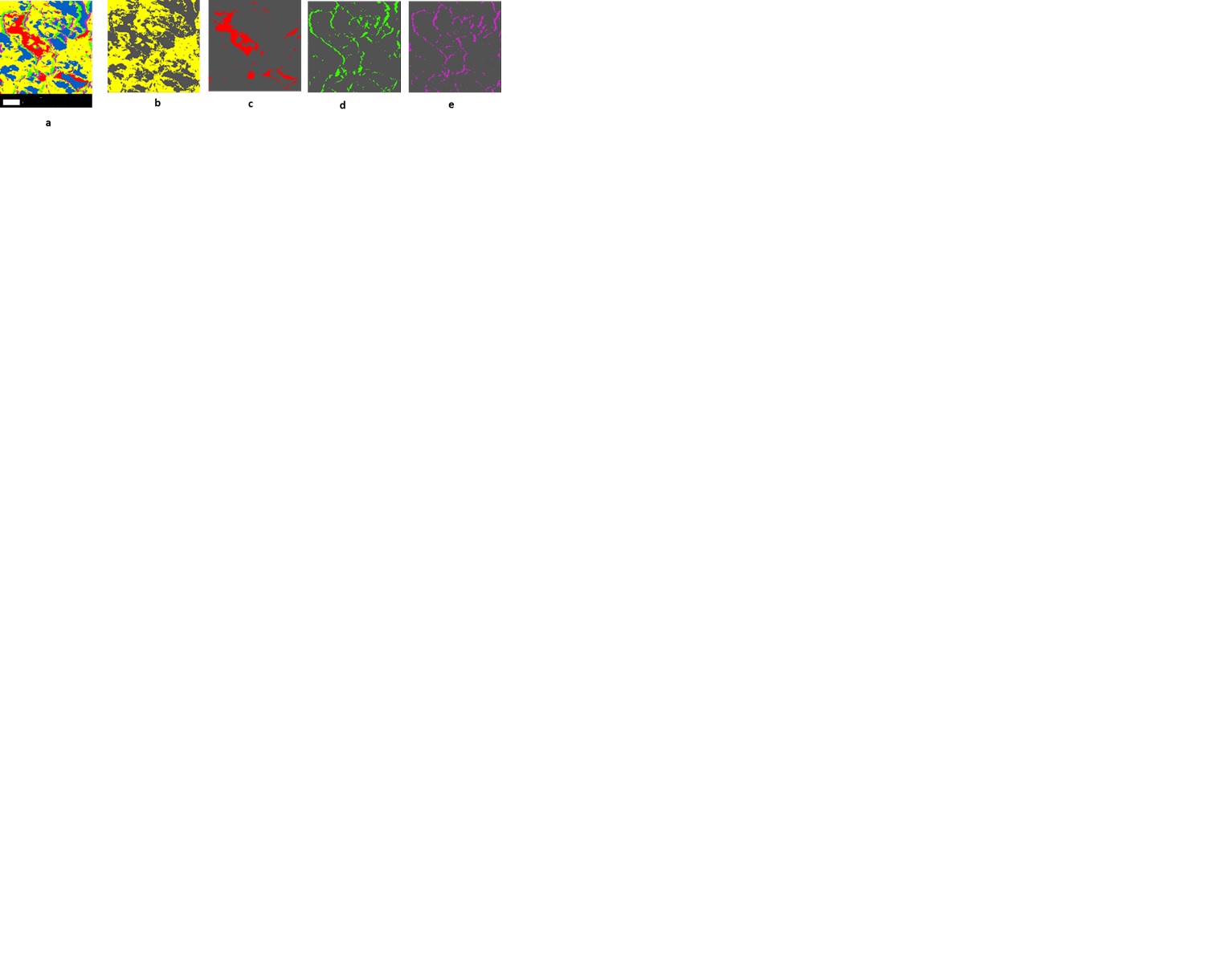


**4µm**

**Figure S2. Cluster indices map of Ca (a) and individual cluster images (b-e) of an NTR 800-nm thin section (22 µmx22 µm). Blue represents empty spaces and areas with high optical density. NTR represents no-till, complex crop rotation. Individual cluster images with different colors represent the regions with similar spectral properties.**


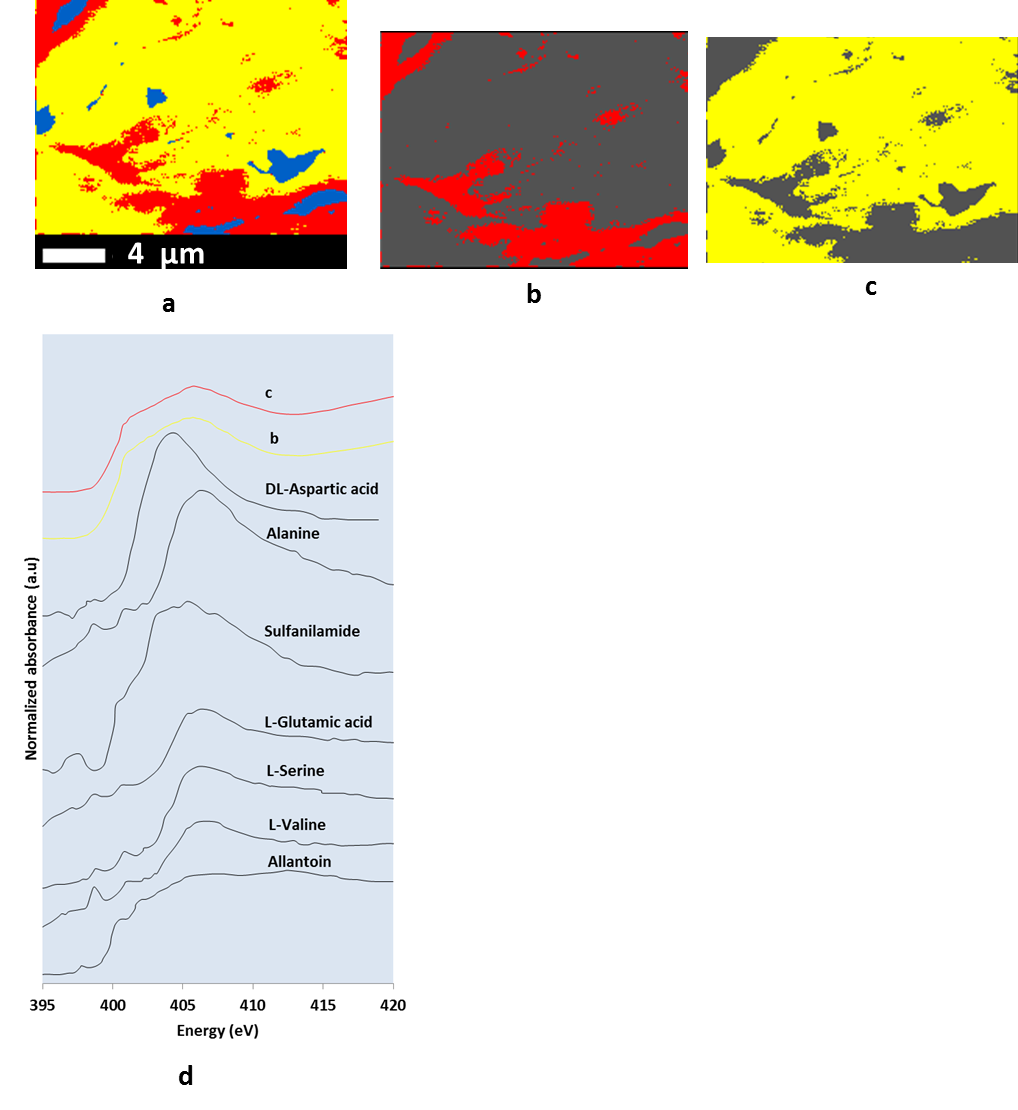


**Figure S3. Cluster indices map of N (a) and individual cluster images (b-c) of an NTR 100-nm thin section (20 µmx15 µm). Nitrogen K-edge (1s transitions) NEXAFS spectra (clusters b and c) and re-created N standard spectra^1^ (d). Blue represents empty spaces and areas with high optical density. NTR represents no-till, complex crop rotation. Individual cluster images with different colors represent the regions with similar spectral properties.**

**
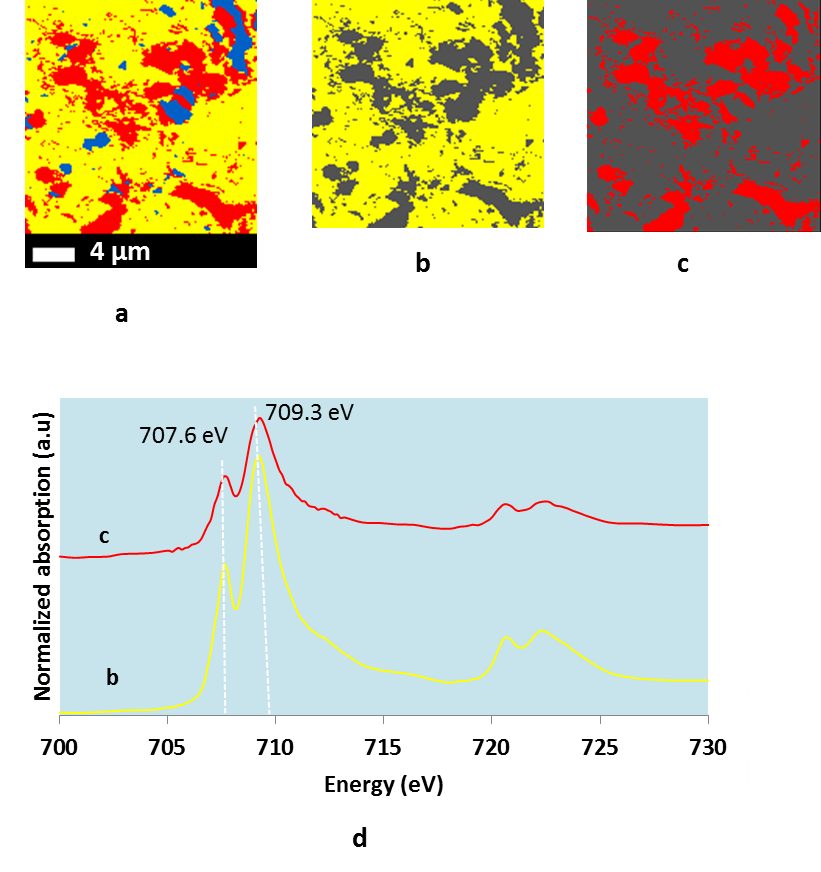
**

Figure S4. Cluster indices map of Fe (a), individual cluster images (b-c), Fe L-edge (2p transitions) NEXAFS spectra (d) of an NTR 800-nm thin section (22 µm x 22 µm). Blue represents empty spaces and areas with high optical density. NTR represents no-till, complex crop rotation. Individual cluster images with different colors represent the regions with similar spectral properties.


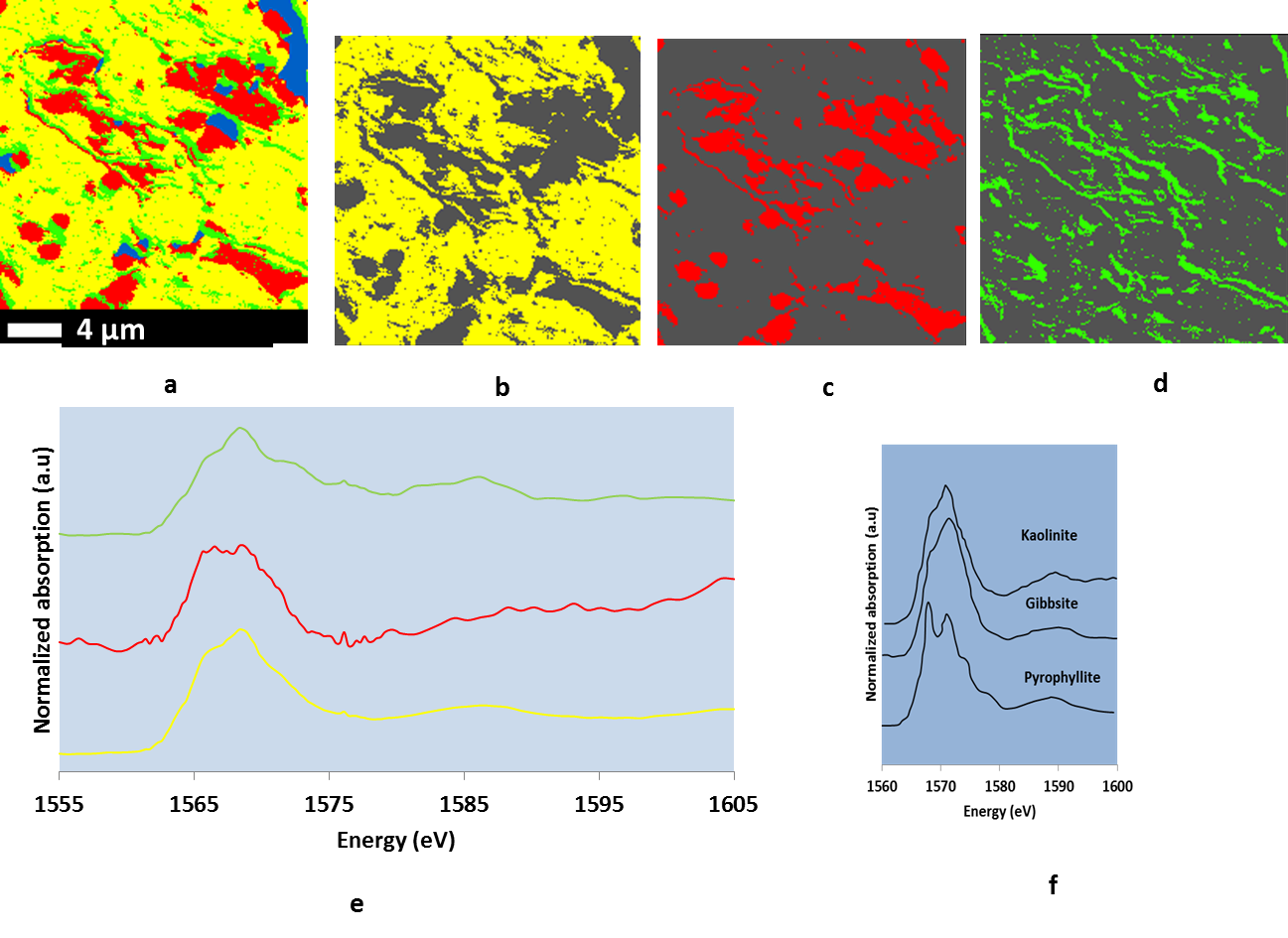


**d**

**c**

**b**

**Figure S5. Aluminum cluster indices map (a), individual cluster images (b-d), and Al K-edge (1s transitions) NEXAFS spectra (e) of an NTR-800 nm thin section (22 µm x 22 µm). Blue represents empty spaces and areas with high optical density. NTR represents no-till, complex crop rotation. Spectral shapes (e) indicate the presence of kaolinite and gibbsite-like minerals. Cluster c (indicated by the red spectra) indicated the presence of pyrophyllite-like minerals Re-created standard spectra of kaolinite and gibbsites^2^ are shown (f). Individual cluster images with different colors represent the areas with similar spectral properties.**


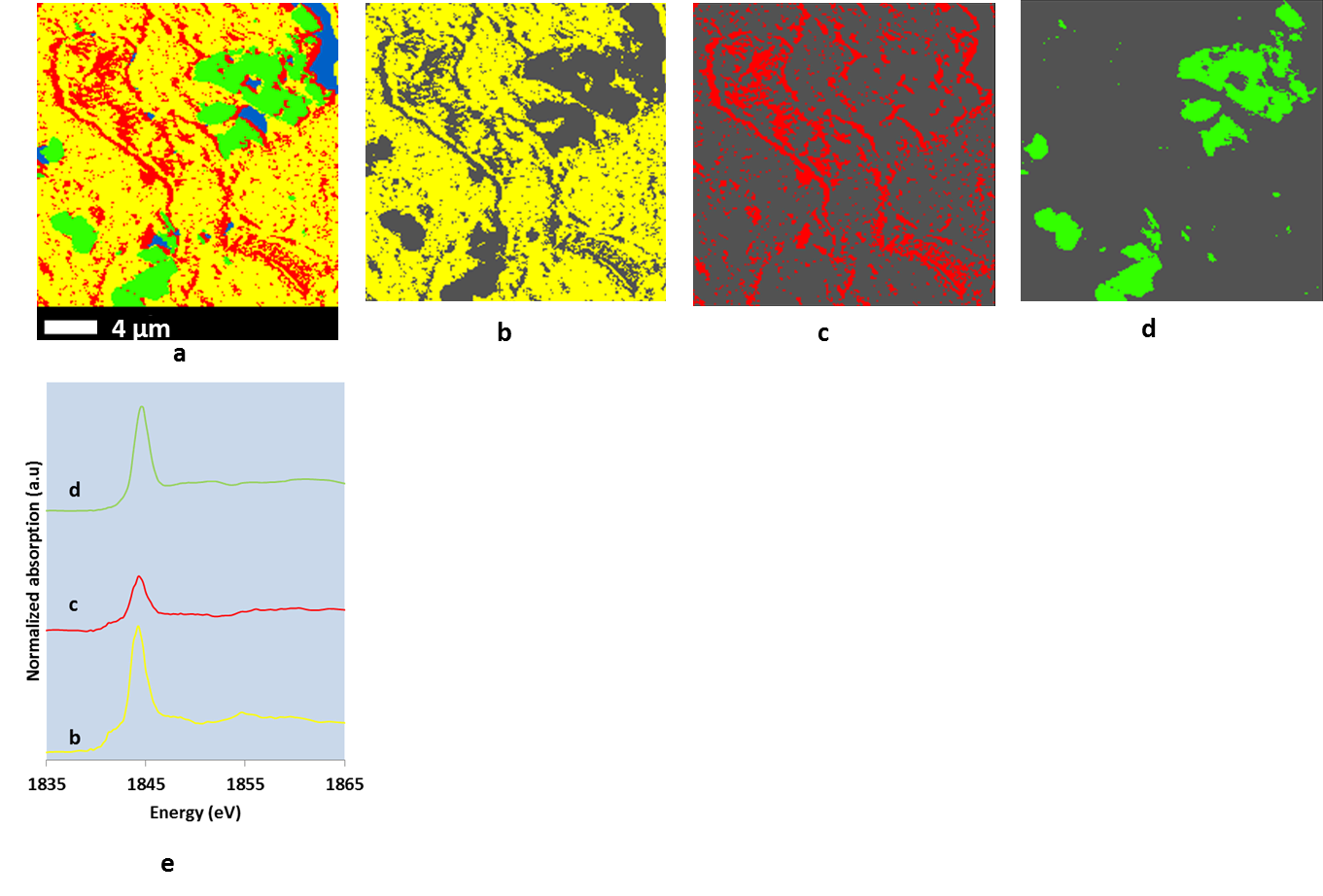


Figure S6. Cluster indices map of Si (a), individual cluster images (b-d), and Si K-edge (1s transitions) NEXAFS spectra (e) of an NTR 800-nm thin section (22 µm x 22 µm). Blue represents empty spaces and areas with high optical density. NTR represents no-till, complex crop rotation. Individual cluster images with different colors represent the areas with similar spectral properties.


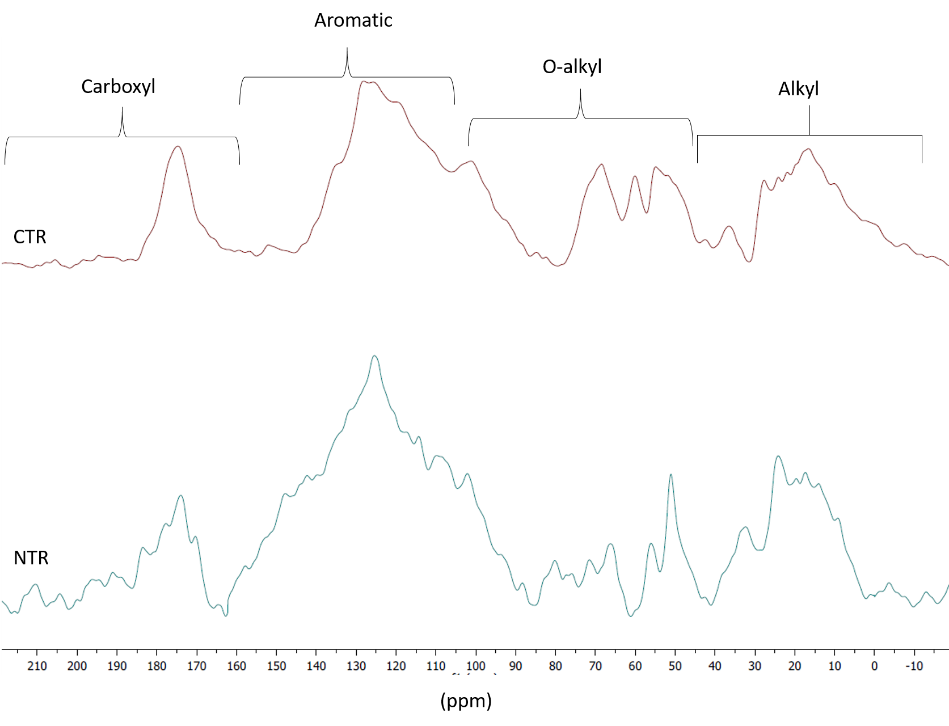


Figure S7. ^13^C-NMR spectra of humic acid extracted from soil microaggregates (150-250 µm fraction) of NTR and CTR. NTR represents no-till, complex crop rotation. CTR represents conventional till, complex crop rotation.


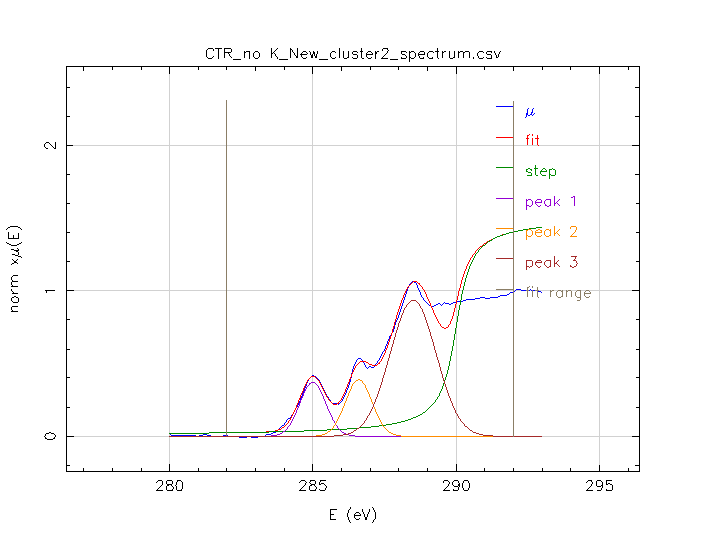


Figure S8. An example of Gaussian peak fitting (using ATHENA) representing C K-edge (1s transitions) NEXAFS spectrum of cluster g of CTR-100 nm thin section. Peak 1 indicates aromatic-C, 2 indicates ketonic C, and 3 indicates carboxylic C. CTR represents conventional till, complex crop rotation.

**Supplementary Tables**

Table S1. Percentages (%) of different C functional groups (based on Gaussian peak fitting) of C K-edge (1s transitions) NEXAFS spectra, representing NTR 100-nm thin section (Fig. 1e).

|  | **Cluster b** | **Cluster c** | **Cluster d (preserved feature)** |
| --- | --- | --- | --- |
| Aromatic | 17.9 (±3.6) | - | 20.7 (±4.1) |
| Ketonic/Phenolic | - | - | 24.8 (±4.1) |
| Aliphatic | 31.4 (±3.6) | 23(±6.4) | - |
| Carboxylic | 66.7 (±3.7) | 77(±6.5) | 54.5 (±4.0) |
| R-factor, red. χ2 | 0.03, 0.02 | 0.05, 0.02 | 0.04, 0.02 |

**NTR represents no-till, complex crop rotation. Red. χ2= =∑ [(fit-data)/ ε]^2^/(N_data_- N_components_). Here ε is the estimated uncertainty in the normalized NEXAFS data. R-factor*= Σ(μ_exp_ – μ_model_)^2^ / Σ(μ_exp_)^2^.* The total percentage was constrained to be 100% in all fits. The uncertainty in parenthesis are 1sigma uncertainties calculated by scaling the diagonal elements of the covariance matrix by the square root of reduced chi-square with the assumption that each fit is a good fit. Uncertainties associated in the percentages listed for each standard component are <10%.**

Table S2. Percentages (%) of different C functional groups (based on Gaussian peak fitting) of C K-edge (1s transitions) NEXAFS spectra, representing CTR 100-nm thin section (Fig.1k).

|  | **Cluster g** | **Cluster h** | **Cluster i** | **Cluster j**  **(preserved feature)** |
| --- | --- | --- | --- | --- |
| Aromatic | 19.2 (±3.2) | 24.9 (±2.8) | 20.8 (±3.2) | 35.3 (±2.2) |
| Ketonic | 23.2 (±3.3) | 22.4 (±2.9) | 19.3 (±3.3) | 28 (±2.2) |
| Carboxylic | 57.6 (±3.3) | 52.7 (±3) | 59.9 (±3.4) | 36.7 (±2.2) |
| R-factor, red. χ2 | 0.04, 0.02 | 0.03, 0.02 | 0.04, 0.02 | 0.03, 0.015 |

**CTR represents conventional till, complex crop rotation. Red. χ2= =∑ [(fit-data)/ ε]^2^/(N_data_- N_components_). Here ε is the estimated uncertainty in the normalized NEXAFS data. R-factor*= Σ(μ_exp_ – μ_model_)^2^ / Σ(μ_exp_)^2^.* The total percentage was constrained to be 100% in all fits. The uncertainty in parenthesis are 1sigma uncertainties calculated by scaling the diagonal elements of the covariance matrix by the square root of reduced chi-square with the assumption that each fit is a good fit. Uncertainties associated in the percentages listed for each standard component are <10%.**

**Table S3. Percentages (%) of Ca species of Ca L-edge (2p transitions) NEXAFS spectra representing individual cluster images of an NTR 100-nm thin section, determined by linear combination fitting (Fig. 3e).**

| **Cluster** | **Composition** | **R-factor, red. χ2** |
| --- | --- | --- |
| b | Hydrous calcium dihydrogen phosphate:57.6 (±1.7) %  Adsorbed Ca_eps: 42.4 (±1.7) % | 0.008, 0.29 |
| d (preserved feature) | Hydrous calcium dihydrogen phosphate:91.9(±6.3) %  Calcium sulphate: 8.1 (±6.3) % | 0.03, 2.6 |

**NTR represents no-till, complex crop rotation. Data are only shown for significant fits. Red. χ2= =∑ [(fit-data)/ ε]^2^/(N_data_- N_components_). Here ε is the estimated uncertainty in the normalized NEXAFS data. R-factor*= Σ(μ_exp_ – μ_model_)^2^ / Σ(μ_exp_)^2^.* The total percentage was constrained to be 100% in all fits. The uncertainty in parenthesis are 1sigma uncertainties calculated by scaling the diagonal elements of the covariance matrix by the square root of reduced chi-square with the assumption that each fit is a good fit. Uncertainties associated in the percentages listed for each standard component are <10%.**

| **Cluster** | **Composition** | **R-factor, red. χ2** |
| --- | --- | --- |
| g | Calcium sulphate: 36.9 (±6.1) %  Hydrous calcium dihydrogen phosphate:63.1 (±6.1) % | 0.026, 0.79 |
| j (preserved feature) | Calcium sulphate: 44.6 (±2.1) %  Hydrous calcium dihydrogen phosphate: 39.9 (±1.7) %  Calcite: 15.5 (±1.3)% | 0.004, 0.06 |

**Table S4. Percentages (%) of Ca species of Ca L-edge (2p transitions) NEXAFS spectra representing individual cluster images of a CTR 100-nm thin section, determined by linear combination fitting (Fig. 3k).**

**CTR represents conventional till, complex crop rotation. Data are only shown for significant fits. Red. χ2= =∑ [(fit-data)/ ε]^2^/(N_data_- N_components_). Here ε is the estimated uncertainty in the normalized NEXAFS data. R-factor*= Σ(μ_exp_ – μ_model_)^2^ / Σ(μ_exp_)^2^.*The total percentage was constrained to be 100% in all fits. The uncertainty in parenthesis are 1sigma uncertainties calculated by scaling the diagonal elements of the covariance matrix by the square root of reduced chi-square with the assumption that each fit is a good fit. Uncertainties associated in the percentages listed for each standard component are <10%.**

| **Cluster** | **Composition** | **R-factor, red. χ2** |
| --- | --- | --- |
| b | Goethite: 21.6 (±3.7)%;  Ferric phosphate: 31.1(±1.9)%  Maghemite: 13.2 (±5.8)%  Magnetite 34.1 (±7.2)% | 0.005,1.18 |
| c | Goethite: 27.6 (±1.3)%  Fe(II) hydroxycarbonate 22.8(±3.3)%  Maghemite: 49.6 (±3.6)% | 0.021, 0.89 |
| d | Maghemite 87.1(±0.3)%  Ferric phosphate 12.9(±0.3)% | 0.009, 0.38 |

Table S5. Percentages (%) of Fe species of Fe L-edge (2p transitions) NEXAFS spectra representing individual cluster images of an NTR 100-nm thin section, determined by linear combination fitting (Fig. 4e).

**CTR represents conventional till, complex crop rotation. Data are only shown for significant fits. Red. χ2= =∑ [(fit-data)/ ε]^2^/(N_data_- N_components_). Here ε is the estimated uncertainty in the normalized NEXAFS data. R-factor*= Σ(μ_exp_ – μ_model_)^2^ / Σ(μ_exp_)^2^.*The total percentage was constrained to be 100% in all fits. The uncertainty in parenthesis are 1sigma uncertainties calculated by scaling the diagonal elements of the covariance matrix by the square root of reduced chi-square with the assumption that each fit is a good fit. Uncertainties associated in the percentages listed for each standard component are <10%.**

| **Cluster** | **Composition** | **R-factor, red. χ2** |
| --- | --- | --- |
| g | Maghemite 42.1 (±1.3) %;  Magnetite 57.9 (±1.3) % | 0.02, 0.57 |
| h | Magnetite 57.8 (±3.2) %  Ferric phosphate 27.4 (±0.5) %  Ferrihydrite 14.8 (±3.2) % | 0.009, 1.27 |
| i | Maghemite 55.5 (±0.1) %  Magnetite 45.5 (±0.1) % | 0.017, 0.36 |

**Table S6. Percentages (%) of Fe species of Fe L-edge (2p transitions),** **NEXAFS spectra representing individual cluster images of an CTR 100-nm thin section, determined by linear combination fitting (Fig. 4k).**

**CTR represents conventional till, complex crop rotation. Data is only shown for significant fits. Red. χ2= =∑ [(fit-data)/ ε]^2^/(N_data_- N_components_). Here ε is the estimated uncertainty in the normalized NEXAFS data. R-factor*= Σ(μ_exp_ – μ_model_)^2^ / Σ(μ_exp_)^2^.* The total percentage was constrained to be 100% in all fits. The uncertainty in parenthesis are 1sigma uncertainties calculated by scaling the diagonal elements of the covariance matrix by the square root of reduced chi-square with the assumption that each fit is a good fit. Uncertainties associated in the percentages listed for each standard component are <10%.**

**Table S7. Peak assignment for carbon K-edge (1s transitions) NEXAFS spectra.**

| **Form of C** | **Transition** | **Peak energy (eV)** | **References** |
| --- | --- | --- | --- |
| Alkylated to carbonyl-substituted aromatic C | 1s-π* | 284.9-285.5 | ^3, 4^ |
| Phenolic C | 1s–π* | 285.3-285.7 | ^5^ |
| Ketonic C | 1s–π* | 286.5-286.7 | ^5^ |
| Aliphatic C-H | 1s-3p/ϭ* | 287.1-287.4 | ^6^ |
| Carboxylic C | 1s-π* | 287.7-288.6 | ^6^ |
| Carbonyl C | 1s-3p, ϭ * | 289.3 | ^6^ |
| Carbonates |  | 290-290.5 | ^7^ |

**References**

1. Leinweber, P. *et al*. Nitrogen K-edge XANES-an overview of reference compounds used to identify unknown organic nitrogen in environmental samples. Journal of Synchrotron Radiation 14, 500-511 (2007).

<https://doi.org/10.1107/S0909049507042513>

2. Ildefonse, P. *et al*. Aluminium X-ray absorption near edge structure in model compounds and Earth’s surface minerals. Physics and Chemistry of Minerals 25, 112-121 (1998).

<https://doi.org/10.1007/s002690050093>

3. Brandes, J. A. *et al*. Examining marine particulate organic matter at sub-micron scales using scanning transmission X-ray microscopy and carbon X-ray absorption near edge structure spectroscopy. Mar. Chem. 92, 107-121 (2004).

<https://doi.org/10.1016/j.marchem.2004.06.020>

4. Braun, A. *et al*. Advantages of soft X-ray absorption over TEM-EELS for solid carbon studies-a comparative study on diesel soot with EELS and NEXAFS. Carbon 43, 117-124 (2005).

<https://doi.org/10.1016/j.carbon.2004.08.029>

5. Solomon, D. et al. Micro-and nano-environments of carbon sequestration: Multi-element STXM–NEXAFS spectromicroscopy assessment of microbial carbon and mineral associations. Chem. Geol. 329, 53-73 (2012).

<https://doi.org/10.1016/j.chemgeo.2012.02.002>

6. Lehmann, J. *et al*. Near‐edge X‐ray absorption fine structure (NEXAFS) spectroscopy for mapping nano‐scale distribution of organic carbon forms in soil: Application to black carbon particles. *Global Biogeochem*. *Cycles*. 19, 002435; 10.1029/2004GB002435 (2005).

<https://doi.org/10.1029/2004GB002435>

7. Brandes, J. A., Wirick, S. & Jacobsen, C. Carbon K-edge spectra of carbonate minerals. Journal of synchrotron radiation 17, 676-682 (2010).

<https://doi.org/10.1107/S0909049510020029>
